# Update on Neoadjuvant Regimens for Patients with Operable Oesophageal/Gastrooesophageal Junction Adenocarcinomas and Squamous Cell Carcinomas

**DOI:** 10.1007/s11912-017-0559-8

**Published:** 2017-02-17

**Authors:** Samantha J. Cox, Sean M. O’Cathail, Bernadette Coles, Tom Crosby, Somnath Mukherjee

**Affiliations:** 1 Cardiff University, Cardiff, CF10 3XQ UK; 2 Department of Clinical Oncology, Velindre Cancer Centre, Cardiff, UK; 3 Department of Clinical Oncology, Oxford University NHS Trust, Oxford, UK; 4 Library Services, Velindre NHS Trust, Cardiff, UK; 5 MRC/CRUK Oxford Institute for Radiation Oncology, University of Oxford, Oxford, UK

**Keywords:** Neoadjuvant, Oesophagus, Gastrooesophageal junction, Adenocarcinoma, Squamous cell carcinoma, Chemotherapy, Chemoradiotherapy

## Abstract

Survival outcomes following multimodal treatment of operable oesophageal and gastrooesophageal cancer remain disappointingly poor. Although an appreciation of the impact of both tumour location and histological subtype is now shaping the design of clinical trials, there has been a lack of consensus of the optimal neoadjuvant treatment strategy. This update article will review recent advances in the use of both neoadjuvant chemotherapy and chemoradiotherapy. The emerging role of PET imaging to direct appropriate neoadjuvant treatment regimens and the additive benefit of biological agents are also discussed.

## Introduction

Approximately 450,000 new cases of oesophageal cancer are diagnosed each year worldwide, making it the eighth most common cancer [1]. Although squamous cell carcinoma (SCC) is the most common variant, there is geographical variation in the incidence of the two major histological subtypes. The number of patients diagnosed with adenocarcinoma has increased during the last few decades, particularly in Western countries, and is felt to reflect the rise in obesity rates and gastrooesophageal reflux disease [2, 3]. Adenocarcinoma typically affects the lower oesophagus and gastrooesophageal junction (GOJ) and likely represents a separate disease entity to squamous cell tumours, which are more common in the upper and middle third of the oesophagus.

Long-term outcomes for oesophageal cancer remain poor with 5-year overall survival (OS) rates of only around 20%, and the majority of patients treated with curative intent eventually succumb to their disease [4]. Patients deemed to have potentially resectable disease are offered a multimodal approach with either neoadjuvant or perioperative chemotherapy or neoadjuvant chemoradiotherapy. The rationale for perioperative therapy is to treat occult micrometastases, and to increase the likelihood of a complete surgical resection (R0 resection) and pathological complete response (pCR).

Historically studies have included oesophageal, GOJ and gastric tumours of both SCC and adenocarcinoma subtypes. These specific subgroups are important in the management of oesophageal cancer, as reflected by the design of more recent trials. This review will summarise the current evidence for neoadjuvant chemotherapy in both histological subtypes, perioperative chemotherapy in adenocarcinoma of the oesophagus/GOJ, and neoadjuvant chemoradiotherapy (nCRT). The role of PET imaging in guiding neoadjuvant treatment strategies and the use of novel biological agents is also discussed. Table 1 summarises ongoing neoadjuvant trials in oesophageal cancer.

**Table 1:** Summary of ongoing neoadjuvant trials in oesophageal cancer

Trial name and objective	Histology, stage and tumour location	Study design	Treatment arms	Primary endpoint	Secondary endpoints
JCOG 1109 NExT study [24] To establish the superior neoadjuvant treatment regimen in resectable locally advanced oesophageal cancer	SCC, adenosquamous carcinoma or basaloid cell carcinoma; stages IB, II and III (excluding T4); thoracic oesophagus	Phase III, multi-institutional three-arm open-label randomised study Estimated enrolment: 501 patients	A: 2 courses of neoadjuvant cisplatin (80 mg/m^2^, day 1) and 5-fluorouracil (800 mg/m^2^/day, days 1–5), qd21 B: 3 courses of neoadjuvant DCF (docetaxel, 70 mg/m^2^, day 1); (cisplatin, 70 mg/m^2^, day 1; 5-fluorouracil, 750 mg/m^2^/day, days 1–5), qd21 C: Neoadjuvant chemoradiotherapy (41.4 Gy/23 fractions over 5 weeks with 2 courses of cisplatin (75 mg/m^2^, day 1) and 5-fluorouracil (1000 mg/m^2^/day, days 1–4), qd28)	Overall survival	Progression-free survival, % R0 resection, response rate, pathological complete response rate, adverse events
Neo-AEGIS [37] Survival outcomes for perioperative chemotherapy (MAGIC) versus neoadjuvant CRT (CROSS) in resectable oesophageal cancer	Adenocarcinoma; cT2-3 N0-3 M0; oesophagus or gastrooesophageal junction	Phase III multi-centre open-label randomised controlled trial Estimated enrolment: 594 patients	A: 3 courses of neoadjuvant followed by 3 courses of adjuvant ECF/ECX (epirubicin, 50 mg/m^2^, day 1); cisplatin, 60 mg/m^2^ day 1; 5-fluorouracil (200 mg/m^2^/day continuous infusion) or capecitabine (625 mg/m^2^/day, d1–21), qd21 B: Neoadjuvant chemoradiotherapy (41.4 Gy/23 fractions over 5 weeks with weekly carboplatin) (AUC2) and paclitaxel (50 mg/m^2^)	1-, 2- and 3-year survival	Clinical and pathological response rates, tumour regression grade, node-positivity, postoperative histology, disease-free survival, time to treatment failure, toxicity, postoperative complications, health-related quality of life

## Neoadjuvant Chemotherapy in Oesophageal Cancer (both Adenocarcinoma and SCC Histologies)

There has been a lack of international consensus regarding the role of neoadjuvant chemotherapy for oesophageal cancer following results from conflicting large randomised trials. Practice in the UK was largely dictated by the outcomes of the MRC OE02 trial, which compared 2 cycles of cisplatin and 5-fluorouracil followed by surgery to surgery alone, with a 6% improvement in 5-year OS rates (23 vs 17%) [5, 6]. Tumours were smaller, less locally invasive following neoadjuvant chemotherapy, with lower rates of lymph node positivity, and significantly higher rates of R0 resection compared to those in the surgery-alone arm. Nine percent of patients in both arms received neoadjuvant radiotherapy but this did not influence outcomes in subgroup analysis.

In contrast, the RTOG 8911 trial failed to identify any survival advantage or difference in R0 rates for neoadjuvant cisplatin/5-fluorouracil versus surgery alone [7]. Differences with OE02 include the use of three neoadjuvant cycles with a longer time to surgery, a higher dose of cisplatin and the use of postoperative chemotherapy for stable disease/responders (with 52% of patients receiving at least 1 cycle of adjuvant chemotherapy) [8]. Analysis for histological subtype did not identify a difference in survival outcomes, but subsequent analysis of the long-term results did confirm that an objective response to neoadjuvant chemotherapy was associated with significantly better OS than that of non-responders or patients receiving surgery alone [7].

Two recent meta-analyses have confirmed the survival benefit for neoadjuvant chemotherapy over surgery alone [9, 10•]. The first meta-analysis of 17 trials found a survival benefit of 5.1% at 2 years (HR 0.87) (95% CI 0.79–0.96; *P* = 0.005) [9]. Analysis according to histological subtype showed only patients with adenocarcinoma (HR 0.83; 95% CI 0.71–0.95; *P* = 0.01) and not SCC (HR 0.92; 95% CI 0.81–1.04; *P* = 0.18) had a statistically significant improvement in OS with neoadjuvant chemotherapy. This is in contrast to the OE02 trial which did not observe a survival difference according to histological subtype, and neoadjuvant chemotherapy is regarded as an option for both SCC and adenocarcinoma tumours in the UK.

The Cochrane meta-analysis of over 2000 patients with localised, potentially resectable thoracic oesophageal carcinoma found that the use of cisplatin-based neoadjuvant chemotherapy reduced the risk of death by 12% at 5 years (HR 0.88, 95% CI 0.80–0.96; *P* = 0.003) [10•]. Furthermore, R0 resection rates were significantly higher in patients receiving chemotherapy compared to surgery alone, although both local and distant recurrence rates did not significantly differ between the two groups. Whilst preoperative chemotherapy was associated with mortality and toxicity, the authors did not find that the latter equated to a higher rate of postsurgical complications. They noted that significant study heterogeneity exists in terms of the chemotherapy regimens used, provision of adjuvant chemotherapy, and inclusion of patients with tumours of both histological subtypes and also the quality of included trials. The conclusion is that the evidence to date for neoadjuvant chemotherapy is only of moderate quality for thoracic oesophageal cancer.

## Perioperative Chemotherapy for Adenocarcinoma of the Lower Oesophagus and GOJ

The efficacy of perioperative chemotherapy for resectable adenocarcinoma of the lower third of the oesophagus, and GOJ was established in the MAGIC trial [11]. Six cycles of epirubicin, cisplatin and 5-fluorouracil (ECF; 3 cycles administered before and after surgery, qd21) improved both OS and PFS in over 500 patients, with a 5-year survival rate of 36% compared to 23% for surgery alone. Downstaging and R0 rates were also superior with ECF, as were local and failure rates. This was despite 45% of patients in the perioperative chemotherapy arm not receiving any postoperative treatment. Initially only patients with operable gastric carcinoma were eligible for inclusion; in light of the increasing incidence of GOJ adenocarcinoma, patients with lower third oesophageal tumours were later included but only constituted 26% of the population which may limit the applicability of the results to oesophageal cancer patients. Perioperative cisplatin/5-fluorouracil has also shown similar survival benefits and improved R0 rates compared with surgery alone in a smaller trial of predominantly gastrooesophageal adenocarcinoma [12]. Both regimens are now standard treatment options.

Several groups have investigated different combination chemotherapy regimens in phase II studies in an attempt to improve response rates and survival outcomes in locally advanced resectable gastrooesophageal adenocarcinoma. Three cycles of DCF (docetaxel, cisplatin, 5-fluorouracil) before and after surgery resulted in all (*n* = 41) patients achieving a R0 resection with a 10% pCR rate, and 3-year survival of 60% [13]; docetaxel, cisplatin and capecitabine (DCX) has shown similar response rates, with 60% of patients achieving T-stage downstaging and 40% of node-positive patients converting to N0 status [14]. Only around half of patients completed postoperative treatment with both of these taxane-based regimes, most often due to patient choice or fitness.

In the German FLOT4 trial, 714 patients with locally advanced resectable gastric and GOJ tumours were randomised between perioperative FLOT (5-fluorouracil, leucovorin, oxaliplatin and docetaxel, 4 + 4 cycles, qd14) and ECF/ECX. The study demonstrated a 10% increase in pCR rates in favour of the FLOT arm (15.6% versus 5.8%, *P* = 0.015); survival outcomes are awaited [15]. FLOT is a feasible regimen in patients over the age of 65 years, however caution is required in selecting the patients most likely to benefit due to higher toxicity rates compared to those seen with perioperative FLO [16].

Concerns exist regarding increased grade 3/4 chemotherapy-related toxicities, namely neutropenia and mucositis, with taxane-based treatment. However, it is argued that these are manageable with appropriate dose-reductions and prophylactic G-CSF, and may be off-set by the early improvements in dysphagia with such regimens and avoidance of anthracycline-induced cardiotoxicity [13, 14]. Furthermore, postsurgical complications (around 40%) and mortality rates do not appear to be increased; further studies are warranted.

## Neoadjuvant Chemotherapy for Adenocarcinoma of the Lower Oesophagus and GOJ

Extended neoadjuvant chemotherapy as an alternative to perioperative treatment has been proposed as a strategy to avoid the need for postsurgical treatment. Four cycles of ECX (epirubicin, cisplatin and capecitabine) was compared to 2 cycles of cisplatin/5-fluorouracil prior to surgery in the UK OE05 trial which included over 800 patients. Three-year OS rates did not significantly differ at 42 and 39%, respectively (HR 0.92, 95% CI 0.79–1.08; *P* = 0.3017) [17•]. There was a suggestion that ECX improved R0 resection rates and the likelihood of pCR, and although PFS and DFS were both longer in the ECX arm, statistical significance was not met. ECX caused more grade 3/4 chemotherapy-related toxicity, however postsurgical complication and mortality rates did not differ significantly between treatment arms.

The NeoFLOT was a single centre, non-randomised phase II trial where 6 cycles FLOT were administered every 14 days (*n* = 50) prior to surgery in T3/T4 or node positive tumours of the GOJ or stomach [18]. The regimen was well tolerated (main grade 3/4 toxicities included neutropenia, diarrhoea and mucositis), 86% of patients achieved a R0 resection and a 1-year survival rate of 79% was observed. The authors reported that in those achieving either a pCR or near pCR (40%), the majority of patients had intestinal type tumours.

## Neoadjuvant Chemotherapy for SCC

SCC is the predominate histological subtype in Asia where the standard of care for stage II-III disease is neoadjuvant chemotherapy followed by transthoracic oesophagectomy and lymphadenectomy. The JCOG 9907 trial randomised patients to 2 cycles of cisplatin/5-fluorouracil either before or after surgery; 5-year OS rates were 55 versus 43%, respectively (HR 0.73, 95% CI 0.54–0.99, *P* = 0.04), with no significant differences in adverse events between the groups [19, 20].

Interest has recently developed in the potential additive benefit of taxanes following promising Asian phase II feasibility studies in oesophageal SCC and known benefits in head and neck SCC. Perioperative weekly nab-paclitaxel and cisplatin achieved 100% R0 rates and both OS and DFS were significantly longer in patients who were downstaged (63%) compared to those not downstaged [21].

In a trial of 42 patients, impressive 2-year OS and PFS rates of 88 and 75%, respectively were reported with DCF when compared to standard cisplatin/5-fluorouracil [22•]. Over 90% of patients completed at least 2 cycles of neoadjuvant treatment and achieved R0 resection, despite a large proportion of patients (42%) requiring dose reductions after the first cycle. The overall response rate was 64% according to RECIST criteria with a pCR rate of 17%. Despite 83% of patients developing grade 3–4 neutropenia, only 2% had febrile neutropenia; all patients received prophylactic antibiotics but not G-CSF as standard. There were no treatment-related deaths, although overall perioperative complication rates were higher compared to standard cisplatin/5-fluorouracil. A second smaller feasibility study also showed DCF efficacy in this setting [23]; 93% of patients achieved a curative oesophageal resection with a similar toxicity profile, although higher rates of febrile neutropenia were noted at 18%.

The Japanese JCOG1109 NExT three-arm phase III study is comparing neoadjuvant cisplatin/5-fluorouracial to neoadjvuant DCF and to neoadjvuant chemoradiotherapy (concurrent cisplatin/5-fluorouracil, 41.4Gy in 23 fractions over 5 weeks) in SCC of the thoracic oesophagus [24]. The primary end-point is OS, with secondary measures including PFS, pCR/R0 resection rates and toxicities, with the aim of identifying the optimal neoadjuvant treatment strategy in this subgroup.

## Neoadjuvant Chemoradiotherapy (nCRT)

Stage I and II oesophageal cancers are unlikely to require neoadjuvant or perioperative treatment. Indeed, outcomes may be worse because of the morbidity and mortality associated with multimodality treatment [25•]. More locally advanced tumours benefit from neoadjuvant therapy compared to surgery alone [9]. There have been several trials comparing nCRT to surgery alone in oesophageal cancer. However, most of these trials were commenced in the 1980s and 1990s, were underpowered, and there was significant heterogeneity in chemotherapy, radiation doses and surgical technique [26–31]. Unsurprisingly results have been mixed. A 2003 meta-analysis of these trials concluded that OS at 3 years was improved (OR 0.66, CI 0.47–0.92; *P* = 0.016) in the nCRT arms but with the cost of increased all treatment mortality (OR 1.63, CI 0.99–2.68; *P* = 0.053) [32]. A further meta-analysis including trials from 1960 to 2007, concluded that in spite of the significant heterogeneity, nCRT increases 3 year OS versus surgery alone with a number needed to treat (NNT) of 10. There was an increased morbidity and mortality with a number needed to harm (NNH) of 25 [33]. Therefore, on balance, the benefit outweighs the risk for appropriately selected patients.

The German POET study, which sought to establish the superiority of chemoradiation over chemotherapy, closed early due to poor accrual and was significantly underpowered as a result [34]. Patients with locally advanced adenocarcinoma of the EGJ were randomised to either neoadjuvant cisplatin/5-fluorouracil chemotherapy over 15 weeks followed by surgery or 12.5 weeks of induction chemotherapy (cisplatin/5-fluorouracil) followed by nCRT (cisplatin and etoposide, 30Gy in 15 fractions over 3 weeks) and then surgery. Its results showed a non-significant trend in favour of CRT improving survival relative to chemotherapy, but postoperative mortality was higher in the CRT arm.

In 2012, the randomised phase III trial CROSS, established the modern precedent for chemoradiotherapy in the preoperative treatment of oesophageal cancer [35]. Patients were randomly assigned to surgery alone or neoadjuvant treatment with weekly carboplatin (AUC2) and paclitaxel (50 mg/m^2^) given concurrently with 41.4 Gy in 23 fractions of radiation. Median overall survival was more than doubled from 24 to 49 months in patients treated with tri-modality therapy, R0 resections improved as did pCR rates, a benefit which persisted in long-term follow-up [36••]. Adverse events were low, with no increase in postoperative mortality.

The benefit of the CROSS protocol is most pronounced in the squamous cell subgroup. Patients with SCC performed significantly better with a median survival of 81 months compared to 43 months for adenocarcinoma of the oesophagus. Proponents of chemotherapy alone argue that for adenocarcinoma these data are no more persuasive than the MAGIC trial. In the adenocarcinoma subgroup of CROSS, the 5 year OS for CRT and surgery was 47%, compared to 33% in the surgery alone group [36••]; this is similar to the 13% absolute benefit for chemotherapy seen in MAGIC, although the OS rate for surgery only patients in this study was lower at 23% [11]. The ongoing Neo-AEGIS trial, which is randomising patients with operable oesophageal and GOJ adenocarcinoma between the CROSS regimen and perioperative ECX (as in the MAGIC trial), may provide the definitive answer of which regimen to use [37], but until then recommendations are likely to vary.

## Induction Chemotherapy prior to nCRT

Other attempts to improve response rates include induction chemotherapy prior to neoadjuvant chemoradiotherapy. Two cycles of induction oxaliplatin and S-1, followed by concurrent CRT to 46 Gy with the same agents did not improve pCR in a randomised phase II study [38]. Toxicity was significantly increased. These results are supported by phase II trials where 2 cycles of oxaliplatin and 5-fluorouracil were followed by 50.4 Gy using the same chemotherapy without any significant benefit [39, 40].

In summary, induction chemotherapy is likely to only increase toxicity with no discernible benefit, and the choice of concurrent radiosensitizing chemotherapy is likely to be more important.

## Alternative Radiosensitzers

The CROSS regimen chemotherapy used was a deviation from the traditional cisplatin/5-fluorouracil regimen [41]. Weekly low dose carboplatin and paclitaxel were novel choices which proved exceptionally well tolerated in CROSS. Previous attempts to incorporate taxanes into chemoradiotherapy regimes have had varying results. Docetaxel and paclitaxel both show activity in phase I/II settings but may increase toxicity, possibly unacceptably [42–44]. The triple agent arm of the NExT trial which includes docetaxel will likely answer the question of whether additional taxanes are useful and tolerable.

Oxaliplatin with capecitabine is more active in the metastatic setting than cisplatin with 5-fluorouracil, has a more favourable toxicity profile and is easier to administer [45]. Recent results from the NEOSCOPE study are instructive here. This phase II trial randomised patients following induction chemotherapy with oxaliplatin and capecitabine to concurrent chemoradiation to 45 Gy, with either two weekly oxaliplatin-capecitabine or weekly carboplatin and paclitaxel [46]. Only the carboplatin/paclitaxel arm passed the prespecified threshold of pCR of 35%, and will now be used in a phase III setting in comparison with neoadjuvant chemotherapy.

A retrospective comparison of 215 patients treated in a single institution showed that carboplatin/paclitaxel was associated with a better overall survival when compared to cisplatin/irinotecan, cisplatin/5-fluorouracil or cisplatin/5-fluorouracil/cetuximab [47]. On balance, where nCRT is to be used, current data favours the use of weekly carboplatin and paclitaxel based regimen, based on its efficacy and toxicity profile.

## Toxicity of Neoadjuvant Therapy

Concerns over the added acute toxicity of nCRT compared to chemotherapy alone are legitimate. As such factors including patient age, performance status and comorbidities should be carefully considered when selecting neoadjuvant therapy. Rigorous radiotherapy quality assurance is now a standard feature in trials which together with advances in the management of treatment-related toxicities, improvements in postoperative care and the centralisation of surgery to high-volume centres, have improved the outlook for cancer patients treated with multimodality therapy [48]. Data from the Netherlands pre- and postcentralisation of surgery showed that R0 rates improved by 5%, mortality fell from 12 to 4% and overall survival at 2 years increased from 38 to 54% [49]. The complicated, multidisciplinary care of the patient group means that institutional differences in surgical technique, wound management and intensive care protocols make comparisons between patient groups within trials difficult. However, this highlights that perhaps the severity of complications should be reported as well as overall incidence.

A Scandinavian study randomised 181 patients to either neoadjuvant CRT or chemotherapy alone (cisplatin/5-fluorouracil +/− 40 Gy concomitant radiotherapy) with the purpose of comparing postresection morbidity and mortality [50•]. They concluded that CRT did not increase the chances of complication, but any complication was more likely to be severe. CROSS showed no increase in incidence of complications in the CRT group, but they did not comment on severity [35].

A small substudy of 40 patients enrolled in a randomised phase III trial comparing chemotherapy and CRT examined cardiac function. They found evidence of acute negative effects on systolic and diastolic function, as recorded on echocardiography and *N*-terminal Pro-B-Type Natriuretic peptide, in patients undergoing CRT [51]. The clinical relevance of this needs prospective evaluation but shows clear physiological differences between both regimes.

## PET-Directed Neoadjuvant Strategies

As well as the obvious need to stratify future oesophageal studies by tumour subtype, there is a need for other predictive markers to assess response to treatment. To date the most studied, for both aspects, has been 18F-flourodeoxyglucose PET-CT.

MUNICON-I trial showed that PET response can be used to direct further treatment in distal adenocarcinoma of the oesophagus [52]. A >35% decrease in SUV indicated metabolic response and assessment was done 14 days after day 1 of cisplatin/5-fluorouracil. Responders continued with treatment but non-responders proceeded directly to surgery. The results showed that metabolic responders had not reached median OS at the time of reporting, whereas non-responders had a median OS of 25.8 months. This implies that patients who are not responding to standard induction chemotherapy, according to PET criteria, can discontinue neoadjuvant therapy and proceed to surgery without compromise.

The follow-up trial, MUNICON-II, attempted to clarify the role of salvage nCRT for patients failing to demonstrate a PET-response following 14 days of cisplatin/5-fluorouracil based induction chemotherapy. Metabolic responders (*n* = 33) continued on 3 months of neoadjuvant chemotherapy prior to surgical resection whilst the non-responders (*n* = 23) received 32 Gy in twice daily 1.6 Gy fractions, 10 fractions per week, with either concurrent cisplatin or 5-fluorouracil [53]. The R0 resection rate was 82% in the PET responders but a lower than expected rate of 70% was observed in the non-responders, resulting in this primary study endpoint not being met. One-year PFS rate was significantly higher in responders (74 vs 57%, *P* = 0.035) although overall survival was similar at around 80%. The authors argue that although salvage nCRT resulted in a major histopathological response rate in 26% of PET non-responders, long-term clinical outcomes were not improved. They suggest the aggressive tumour biology of this group is responsible for these results and that studies exploring alternative approaches, including the addition of systemically active drugs, are warranted [54]. A reduction in the metabolic activity of a tumour, defined by a 35% decrease in FDG uptake compared to baseline, at day 14 of neoadjuvant chemotherapy has been prospectively validated as prognostic biomarker [55]. PET responders had a 3-year survival of 70% compared to 35% for non-responders. In an exploratory substudy within a phase II study of induction cisplatin and irinotecan, an SUV reduction of >35% again predicted for improved outcomes; higher pCR, increased R0, longer PFS and improved OS (40.2 versus 25.5 months) [56].

Data supporting the use of PET during nCRT is less convincing, potentially due to false positive results as a consequence of treatment-induced inflammation or false negatives due to small volume occult disease [57]. Within the CROSS trial, a substudy of 100 patients had a repeat PET scan 14 days after commencing nCRT, the results of which were correlated to the histopathological response as defined by the Mandard score (no or <10% viable tumour) on the resection specimen. A median SUV decrease of 30.9% was observed in histopathological responders (*n* = 64) versus 1.7% for non-responders (*n* = 36) (*P* = 0.001). Although the sensitivity of PET in correctly identifying patients with a histopathological response was 91% using the predefined cut-off SUV value of 0% (signifying any decrease in SUV as a positive test result), the specificity was only 50% [58]. On the basis of these results, FDG-PET cannot currently be recommended to accurately identify patients who are not responding nCRT after 2 weeks of treatment. An excellent overview of the literature by Omloo et al. points out that standardisation of protocols and minimising inter-institutional differences is needed before PET-guided treatment can be routinely adopted for chemoradiation patients [59].

## Biological Agents with Chemotherapy and Chemoradiotherapy

The use of targeted therapies in combination with chemotherapy in locally advanced gastrooesophageal adenocarcinoma remains investigational. Perioperative cetuximab/5-fluorouracil did not demonstrate efficacy with 71% of patients (*n* = 64) classified as non-responders [60]. Similarly, the addition of neoadjuvant panitumumab to ECX did not improve the rates of downstaging or pCR in an AIO phase II study [61]. Bevacizumab and perioperative ECX (ECX-B) chemotherapy did not improve survival outcomes in patients with resectable lower oesophageal or GOJ adenocarcinoma [62•]. Recently published in abstract form, the phase III ST03 trial showed similar rates in both response to preoperative therapy, R0 resections and 3-year OS (48.9% ECX vs 47.6% ECX + B; OS HR 1.067) but an increased rate of anastomotic leak following oesophago-gastrectomy in patients receiving ECX-B (23 vs 9%).

Trastuzumab with cisplatin/5-fluorouracil chemotherapy is known to improve OS in advanced HER-2 positive gastric and GOJ tumours [63], and phase II studies have assessed its efficacy in locally advanced gastrooesophageal adenocarcinoma. pCR rates of more than 20% were achieved using perioperative trastuzumab in combination with FLOT, with 93% of patients (*n* = 45) achieving a R0 resection; adverse events were as expected with this regimen [64]. Perioperative trastuzumab and XELOX may also have activity with a reported 18 month DFS of 71% [65].

Further modification of chemoradiotherapy with biological agents appears attractive given their success in the neighbouring anatomical site of head and neck [66], in particular agents that target the epidermal growth factor receptor (EGFR). A phase Ib/II trial of induction cisplatin, docetaxel and cetuximab followed by 45 Gy with cisplatin and cetuximab showed acceptable toxicity with 86% of patient completing therapy and a pCR rate of 68% and a phase III trial is ongoing [67]. A novel EGFR inhibitor, nimotuzumab, has been trialled with cisplatin and CRT (cisplatin and 5-fluorouracil) [68]. In an almost exclusive SCC population, it did not meet its primary endpoint of endoscopic CR but the addition of nimotuzumab did significantly improve a secondary endpoint of combined endoscopic CR and pCR (62.3 vs 33.3%). This regimen may be taken forward for phase III testing. The addition of panitumumab to cisplatin and docetaxel augmented radiation resulted in a pCR rate of 32.8% however, the toxicity profile was significant with 23% having grade 4 + toxicity [69]. A small phase II study of 33 patients adding bevacizumab to cisplatin and irinotecan modified radiation given in the neoadjuvant setting found that although the regime was tolerable there was no suggestion of improvement in PFS, OS or pCR [70]. The regimen was deemed unsuitable for taking forward to the phase III setting.

## Conclusions

Rapid developments over the last 15 years in the management of oesophageal cancer have left many clinicians uncertain in how best to proceed. Ongoing trials, organised according to histological subtype and anatomical location, will help to unravel the uncertainty caused by the significant heterogeneity of older data.

In summary, the authors would recommend several key principles of management. Early stage I do tumours do not benefit from neoadjuvant therapy and should proceed directly to surgery; locally advanced tumours will benefit. In SCC, both definitive CRT as well as tri-modality therapy consisting of nCRT and surgery are considered as standard options. Both neoadjuvant chemotherapy and neoadjuvant CRT are treatment options for oesophageal adenocarcinoma, however, it may be particularly useful to consider tri-modality treatment for bulky adenocarcinomas where there is a priori concern about the ability to achieve an R0 margin. In other situations, neoadjuvant chemotherapy alone can be considered an acceptable alternative. Decision making may also not purely be about tumour factors. Baseline patient factors may also favour one form of neoadjuvant therapy over another, for example previous “in field” radiotherapy for another condition may persuade a clinician to use chemotherapy alone. Given the complex nature of care leading to any given outcome, in particular survival, we would also recommend institutions regularly audit their practice to ensure a patient-centred, evidence-based approach.

## References

[1] Cancer Incidence and Mortality Worldwide: IARC CancerBase No. 11 [Internet] [database on the Internet]. International Agency for Research on Cancer; 2013. 2013. Available from: http://globocan.iarc.fr. Accessed Dec 2013.

[2] Pohl H, Sirovich B, Welch HG (2010). Esophageal adenocarcinoma incidence: are we reaching the peak?. Cancer epidemiology, biomarkers & prevention : a publication of the American Association for Cancer Research, cosponsored by the American Society of Preventive Oncology.

[3] Vizcaino AP, Moreno V, Lambert R, Parkin DM (2002). Time trends incidence of both major histologic types of esophageal carcinomas in selected countries, 1973-1995. Int J Cancer.

[4] Oesophageal Cancer Statistics. Cancer Research UK. http://www.cancerresearchuk.org/health-professional/cancer-statistics/statistics-by-cancer-type/oesophageal-cancer. Accessed 26 May 2016.

[5] Medical Research Council Oesophageal Cancer Working G (2002). Surgical resection with or without preoperative chemotherapy in oesophageal cancer: a randomised controlled trial. Lancet.

[6] Allum WH, Stenning SP, Bancewicz J, Clark PI, Langley RE (2009). Long-term results of a randomized trial of surgery with or without preoperative chemotherapy in esophageal cancer. J Clin Oncol.

[7] Kelsen DP, Winter KA, Gunderson LL, Mortimer J, Estes NC, Haller DG (2007). Long-term results of RTOG trial 8911 (USA Intergroup 113): a random assignment trial comparison of chemotherapy followed by surgery compared with surgery alone for esophageal cancer. J Clin Oncol.

[8] Kelsen DP, Ginsberg R, Pajak TF, Sheahan DG, Gunderson L, Mortimer J (1998). Chemotherapy followed by surgery compared with surgery alone for localized esophageal cancer. N Engl J Med.

[9] Sjoquist KM, Burmeister BH, Smithers BM, Zalcberg JR, Simes RJ, Barbour A (2011). Survival after neoadjuvant chemotherapy or chemoradiotherapy for resectable oesophageal carcinoma: an updated meta-analysis. Lancet Oncol.

[10] Kidane B, Coughlin S, Vogt K, Malthaner R (2015). Preoperative chemotherapy for resectable thoracic esophageal cancer. Cochrane Database Syst Rev.

[11] Cunningham D, Allum WH, Stenning SP, Thompson JN, Van de Velde CJ, Nicolson M (2006). Perioperative chemotherapy versus surgery alone for resectable gastroesophageal cancer. N Engl J Med.

[12] Ychou M, Boige V, Pignon JP, Conroy T, Bouché O, Lebreton G (2011). Perioperative chemotherapy compared with surgery alone for resectable gastroesophageal adenocarcinoma: an FNCLCC and FFCD multicenter phase III trial. Journal of clinical oncology : official journal of the American Society of Clinical Oncology.

[13] Ferri LE, Ades S, Alcindor T, Chasen M, Marcus V, Hickeson M (2012). Perioperative docetaxel, cisplatin, and 5-fluorouracil (DCF) for locally advanced esophageal and gastric adenocarcinoma: a multicenter phase II trial. Ann Oncol.

[14] Thuss-Patience PC, Hofheinz RD, Arnold D, Florschutz A, Daum S, Kretzschmar A (2012). Perioperative chemotherapy with docetaxel, cisplatin and capecitabine (DCX) in gastro-oesophageal adenocarcinoma: a phase II study of the Arbeitsgemeinschaft Internistische Onkologie (AIO)^†^. Ann Oncol.

[15] Pauligk C, Tannapfel A, Meiler J, Luley KB, Kopp HG, Homann N et al. Pathological response to neoadjuvant 5-FU, oxaliplatin, and docetaxel (FLOT) versus epirubicin, cisplatin, and 5-FU (ECF) in patients with locally advanced, resectable gastric/esophagogastric junction (EGJ) cancer: Data from the phase II part of the FLOT4 phase III study of the AIO. J Clin Oncol. 2015.

[16] Lorenzen S, Pauligk C, Homann N, Schmalenberg H, Tauchert F, Jager E et al. Feasibility of perioperative chemotherapy with infusional 5-FU, leucovorin, and oxaliplatin with or without docetaxel, in elderly patients with locally advanced esophagogastric cancer. J Clin Oncol Conf. 2012;30(15 SUPPL. 1). 10.1038/bjc.2012.588 PMC3593547 23322206

[17] Cunningham D, Langley R, Nankivell M, Blazeby J, Griffin M, Crellin A (2015). Neoadjuvant chemotherapy for resectable oesophageal and junctional adenocarcinoma: Results from the UK medical research council randomised OEO5 trial (ISRCTN 01852072). Ann Oncol.

[18] Schulz C, Kullmann F, Kunzmann V, Fuchs M, Geissler M, Vehling-Kaiser U (2015). NeoFLOT: Multicenter phase II study of perioperative chemotherapy in resectable adenocarcinoma of the gastroesophageal junction or gastric adenocarcinoma—Very good response predominantly in patients with intestinal type tumors. Int J Cancer.

[19] Hirao M, Ando N, Tsujinaka T, Udagawa H, Yano M, Yamana H (2011). Influence of preoperative chemotherapy for advanced thoracic oesophageal squamous cell carcinoma on perioperative complications. Br J Surg.

[20] Ando N, Kato H, Igaki H, Shinoda M, Ozawa S, Shimizu H (2012). A randomized trial comparing postoperative adjuvant chemotherapy with cisplatin and 5-fluorouracil versus preoperative chemotherapy for localized advanced squamous cell carcinoma of the thoracic esophagus (JCOG9907). Ann Surg Oncol.

[21] Yun F, Jiang Y, Chen Q, Zhou X, Huang Z, Mao W (2015). Survival outcomes in patients with locally advanced esophageal squamous cell carcinoma treated with nab-paclitaxel and cisplatin as neoadjuvant chemotherapy. European journal of cancer (Oxford, England : 1990).

[22] Hara H, Tahara M, Daiko H, Kato K, Igaki H, Kadowaki S (2013). Phase II feasibility study of preoperative chemotherapy with docetaxel, cisplatin, and fluorouracil for esophageal squamous cell carcinoma. Cancer Sci.

[23] Yokota T, Hatooka S, Ura T, Abe T, Takahari D, Shitara K (2011). Docetaxel plus 5-fluorouracil and cisplatin (DCF) induction chemotherapy for locally advanced borderline-resectable T4 esophageal cancer. Anticancer Res.

[24] Nakamura K, Kato K, Igaki H, Ito Y, Mizusawa J, Ando N (2013). Three-arm phase III trial comparing cisplatin plus 5-FU (CF) versus docetaxel, cisplatin plus 5-FU (DCF) versus radiotherapy with CF (CF-RT) as preoperative therapy for locally advanced esophageal cancer (JCOG1109, NExT study). Jpn J Clin Oncol.

[25] Mariette C, Dahan L, Mornex F, Maillard E, Thomas PA, Meunier B (2014). Surgery alone versus chemoradiotherapy followed by surgery for stage I and II esophageal cancer: final analysis of randomized controlled phase III trial FFCD 9901. Journal of clinical oncology : official journal of the American Society of Clinical Oncology.

[26] Apinop C, Puttisak P, Preecha N (1994). A prospective study of combined therapy in esophageal cancer. Hepato-Gastroenterology.

[27] Walsh TN, Grennell M, Mansoor S, Kelly A (2002). Neoadjuvant treatment of advanced stage esophageal adenocarcinoma increases survival. Dis Esophagus.

[28] Bosset JF, Gignoux M, Triboulet JP, Tiret E, Mantion G, Elias D (1997). Chemoradiotherapy followed by surgery compared with surgery alone in squamous-cell cancer of the esophagus. N Engl J Med.

[29] Lee JL, Park SI, Kim SB, Jung HY, Lee GH, Kim JH (2004). A single institutional phase III trial of preoperative chemotherapy with hyperfractionation radiotherapy plus surgery versus surgery alone for resectable esophageal squamous cell carcinoma. Ann Oncol.

[30] Burmeister BH, Smithers BM, Gebski V, Fitzgerald L, Simes RJ, Devitt P (2005). Surgery alone versus chemoradiotherapy followed by surgery for resectable cancer of the oesophagus: a randomised controlled phase III trial. Lancet Oncol..

[31] Urba SG, Orringer MB, Turrisi A, Iannettoni M, Forastiere A, Strawderman M (2001). Randomized trial of preoperative chemoradiation versus surgery alone in patients with locoregional esophageal carcinoma. J Clin Oncol.

[32] Urschel JD, Vasan H (2003). A meta-analysis of randomized controlled trials that compared neoadjuvant chemoradiation and surgery to surgery alone for resectable esophageal cancer. Am J Surg.

[33] Wijnhoven BP, van Lanschot JJ, Tilanus HW, Steyerberg EW, van der Gaast A (2009). Neoadjuvant chemoradiotherapy for esophageal cancer: a review of meta-analyses. World J Surg.

[34] Stahl M, Walz MK, Stuschke M, Lehmann N, Meyer HJ, Riera-Knorrenschild J (2009). Phase III comparison of preoperative chemotherapy compared with chemoradiotherapy in patients with locally advanced adenocarcinoma of the esophagogastric junction. J Clin Oncol.

[35] Van Hagen P, Hulshof MCCM, Van Lanschot JJB, Steyerberg EW, Van Berge Henegouwen MI, Wijnhoven BPL (2012). Preoperative chemoradiotherapy for esophageal or junctional cancer. N Engl J Med.

[36] Shapiro J, Lanschot JJ, Hulshof MC, Hagen P, Berge Henegouwen MI, Wijnhoven BP (2015). Neoadjuvant chemoradiotherapy plus surgery versus surgery alone for oesophageal or junctional cancer (CROSS): long-term results of a randomised controlled trial. The Lancet Oncology.

[37] Keegan N, Keane F, Cuffe S, Cunningham M, Ravi N, Lee G, et al. ICORG 10–14: Neo-AEGIS: A randomized clinical trial of neoadjuvant and adjuvant chemotherapy (modified MAGIC regimen) versus neoadjuvant chemoradiation (CROSS protocol) in adenocarcinoma of the esophagus and esophagogastric junction. J Clin Oncol Conf. 2014;32(15 SUPPL. 1).

[38] Yoon DH, Jang G, Kim JH, Kim YH, Kim JY, Kim HR (2015). Randomized phase 2 trial of S1 and oxaliplatin-based chemoradiotherapy with or without induction chemotherapy for esophageal cancer. Int J Radiat Oncol Biol Phys.

[39] Ajani JA, Xiao L, Roth JA, Hofstetter WL, Walsh G, Komaki R (2013). A phase II randomized trial of induction chemotherapy versus no induction chemotherapy followed by preoperative chemoradiation in patients with esophageal cancer. Annals of oncology : official journal of the European Society for Medical Oncology / ESMO.

[40] Alberts SR, Soori GS, Shi Q, Wigle DA, Sticca RP, Miller RC et al. Randomized phase II trial of extended versus standard neoadjuvant therapy for esophageal cancer, NCCTG (Alliance) trial N0849. J Clin Oncol Conf. 2013;31(15 SUPPL. 1).

[41] Tran Vuong TN, Le Prise E, Vauleon E, Boucher E, Audrain O, Raoul JL (2010). Chemoradiotherapy for cancer of the esophagus: contribution of the leucovorin, 5-fluorouracil bolus, and infusion-cisplatin-radiotherapy schedule starting with two neoadjuvant chemotherapy cycles: results from a pilot study. Dis Esophagus.

[42] Ma HB, Di ZL, Wen J, Ke Y, Sun X, Ren J (2015). Prospective, open, multicentre Phase I/II trial to assess safety and efficacy of neoadjuvant radiochemotherapy with docetaxel and cisplatin for esophageal carcinoma. Jpn J Clin Oncol.

[43] Wu S, Chen MY, Luo JC, Wei L, Chen Z. [Comparison between docetaxel plus cisplatin and cisplatin plus fluorouracil in the neoadjuvant chemoradiotherapy for local advanced esophageal squamous cell carcinoma]. Zhonghua Zhong Liu Za Zhi [Chinese J Oncol]. 2012. 10.3760/cma.j.issn.0253-3766.2012.11.017 23291141

[44] Spigel DR, Greco FA, Meluch AA, Lane CM, Farley C, Gray JR (2010). Phase I/II trial of preoperative oxaliplatin, docetaxel, and capecitabine with concurrent radiation therapy in localized carcinoma of the esophagus or gastroesophageal junction. J Clin Oncol.

[45] Cunningham D, Okines AF, Ashley S (2010). Capecitabine and oxaliplatin for advanced esophagogastric cancer. N Engl J Med.

[46] Mukherjee S, Hurt C, Gwynne S, Bateman A, Gollins S, Radhakrishna R, Canham J, Ray R, Grabsch HI, Sharma RA, Maggs R, Hawkins MA, Sebag-Montefiore D, Maughan T, Griffiths G, Crosby T (2016). NEOSCOPE: A randomised Phase II study of induction chemotherapy followed by either oxaliplatin/capecitabine (OXCAP) or carboplatin/paclitaxel (CarPac) based chemoradiation (CRT) as pre-operative regimen for resectable oesophageal adenocarcinoma. Journal of clinical oncology : official journal of the American Society of Clinical Oncology.

[47] Niu NN, Catalano PJ, Enzinger PC, Bueno R, King BL, Martin NE (2015). A retrospective comparison of neoadjuvant chemoradiation therapy regimens for locally advanced esophageal cancer. International Journal of Radiation Oncology Biology Physics.

[48] Chowdhury MM, Dagash H, Pierro A (2007). A systematic review of the impact of volume of surgery and specialization on patient outcome. Br J Surg.

[49] Wouters MW, Karim-Kos HE, le Cessie S, Wijnhoven BP, Stassen LP, Steup WH (2009). Centralization of esophageal cancer surgery: does it improve clinical outcome?. Ann Surg Oncol.

[50] Klevebro F, Johnsen G, Johnson E, Viste A, Myrnas T, Szabo E (2015). Morbidity and mortality after surgery for cancer of the oesophagus and gastro-oesophageal junction: A randomized clinical trial of neoadjuvant chemotherapy vs. neoadjuvant chemoradiation. Eur J Surg Oncol.

[51] Lund M, Alexandersson von Dobeln G, Nilsson M, Winter R, Lundell L, Tsai JA (2015). Effects on heart function of neoadjuvant chemotherapy and chemoradiotherapy in patients with cancer in the esophagus or gastroesophageal junction–prospective cohort pilot study within a randomized clinical trial. Radiat.

[52] Lordick F, Ott K, Krause BJ, Weber WA, Becker K, Stein HJ (2007). PET to assess early metabolic response and to guide treatment of adenocarcinoma of the oesophagogastric junction: the MUNICON phase II trial. Lancet Oncol.

[53] zum Buschenfelde CM, Herrmann K, Schuster T, Geinitz H, Langer R, Becker K (2011). (18) F-FDG PET-guided salvage neoadjuvant radiochemotherapy of adenocarcinoma of the esophagogastric junction: the MUNICON II trial. J Nucl Med.

[54] Lordick F (2011). Molecular imaging for response monitoring in esophageal cancer. Eur J Cancer.

[55] Ott K, Weber WA, Lordick F, Becker K, Busch R, Herrmann K (2006). Metabolic imaging predicts response, survival, and recurrence in adenocarcinomas of the esophagogastric junction. J Clin Oncol.

[56] Ilson DH, Minsky BD, Ku GY, Rusch V, Rizk N, Shah M (2012). Phase 2 trial of induction and concurrent chemoradiotherapy with weekly irinotecan and cisplatin followed by surgery for esophageal cancer. Cancer.

[57] Wu AJ, Goodman KA (2013). Positron emission tomography imaging for gastroesophageal junction tumors. Semin Radiat Oncol.

[58] van Heijl M, Omloo JM, van Berge Henegouwen MI, Hoekstra OS, Boellaard R, Bossuyt PM (2011). Fluorodeoxyglucose positron emission tomography for evaluating early response during neoadjuvant chemoradiotherapy in patients with potentially curable esophageal cancer. Ann Surg.

[59] Omloo JM, van Heijl M, Hoekstra OS, van Berge Henegouwen MI, van Lanschot JJ, Sloof GW (2011). FDG-PET parameters as prognostic factor in esophageal cancer patients: a review. Ann Surg Oncol.

[60] Mariette C, Piessen G, Monterymard C, Pezet D, Ferru A, Baconnier M, Adhoute X, Tavan D, Lepage C, Bouche O (2014). Efficacy and safety of perioperative chemotherapy with 5FU-cisplatin-cetuximab in gastric and gastroeosphageal junction Adenocarcionmas (GGOJA): a single arm multicentre phase II trial (FFCD 0901). Annals of oncology : official journal of the European Society for Medical Oncology / ESMO.

[61] Stahl M, Mihaljevic AL, Moehler M, Kanzler S, Hoehler T, Thuss-Patience PC et al. Perioperative chemotherapy with ECX +/− panitumumab in locally advanced gastroesophageal adenocarcinomas (GEA): A randomized study of the Arbeitsgemeinschaft internistische onkologie and the chirurgische arbeitsgemeinschaft onkologie of the German cancer society. J Clin Oncol Conf. 2015;33(3 SUPPL. 1).

[62] Cunningham D, Smyth E, Stenning S, Stevenson L, Robb C, Allum W (2015). Peri-operative chemotherapy ± bevacizumab for resectable gastro-oesophageal adenocarcinoma: Results from the UK Medical Research Council randomised ST03 trial. European journal of cancer (Oxford, England : 1990).

[63] Bang YJ, Van Cutsem E, Feyereislova A, Chung HC, Shen L, Sawaki A (2010). Trastuzumab in combination with chemotherapy versus chemotherapy alone for treatment of HER2-positive advanced gastric or gastro-oesophageal junction cancer (ToGA): a phase 3, open-label, randomised controlled trial. Lancet.

[64] Hofeinz R, Hegewisch-Becker S, Thuss-Patience P, Kunzmann V, Fuchs M, Graeven U, Homann N, Heinemann V, Pohl M, Tannapfel A, Al-Batran S (2014). HER-FLOT: trastuzumab in combination with FLOT as perioperative treatment for patients with HER2-positive locally advanced esophagogastric adenocarcinoma: a phase II trial of the AIO gastric cancer study group. journal of clinical oncology : official journal of the American Society of Clinical Oncology.

[65] Rivera F, Jimenez-Fonseca P, Alfonso P, Gallego J, Limon M, Alsina M, Lopez-Gomez L, Galan M, Falco E, Manzano J, Gonzalez E, Aranda E, Fernandez E, Jorge M (2015). NEOHX study: Perioperative treatment with trastuzumab in combination with capecitabine and oxaliplatin (XELOX-T) in patients with HER-2 resectable stomach or esophagogastric junction (EGJ) adenocarcinoma—18 m DFS analysis. Journal of clinical oncology : official journal of the American Society of Clinical Oncology.

[66] Bonner JA, Harari PM, Giralt J, Azarnia N, Shin DM, Cohen RB (2006). Radiotherapy plus cetuximab for squamous-cell carcinoma of the head and neck. N Engl J Med.

[67] Ruhstaller T, Pless M, Dietrich D, Kranzbuehler H, von Moos R, Moosmann P (2011). Cetuximab in combination with chemoradiotherapy before surgery in patients with resectable, locally advanced esophageal carcinoma: a prospective, multicenter phase IB/II trial (SAKK 75/06). J Clin Oncol.

[68] Castro G, Skare NG, Andrade CJC, Segalla LGM, De Azevedo SJ, Silva I, Filho FM, Del Grossi Neusquen LP, de Oliveira Berto CR (2014). Chemoradiation with or without nimotuzumab in locally advanced esophageal cancer (LAEC): A randomized phase II study (NICE trial). Journal of clinical oncology : official journal of the American Society of Clinical Oncology.

[69] Reed CE, Decker PA, Schefter TE, Meyers BF, Ferguson MK, Oeltjen AR, Putnam JB, Cassivi SD, Lockhart AC (2012). A phase II study of neoadjuvant therapy with cisplatin, docetaxel, panitumumab plus radiation therapy followed by surgery in patients with locally advanced adenocarcinoma of the distal esophagus. Journal of clinical oncology : official journal of the American Society of Clinical Oncology.

[70] Ilson D, Goodman KA, Janjigian YY, Shah MA, Kelsen DP, Rizk NP, Rusch VW, Jing-Ching Wu A, Campbell J, Capanu M, Bains MS (2012). Phase II trial of bevacizumab, irinotecan, cisplatin, and radiation as preoperative therapy in esophageal adenocarcinoma. Journal of clinical oncology: official journal of the American Society of Clinical Oncology.

